# Body composition analysis using bioelectric impedance in Bissau: reproducibility and level of agreement between two available devices

**DOI:** 10.11604/pamj.2024.48.80.42997

**Published:** 2024-06-28

**Authors:** Lilica Sanca, Stine Byberg, Cipriano Có, Geovane da Costa, Marceano Indami, Luis Rama, Ana Maria Teixeira, Morten Bjerregaard-Andersen, Eugénia Carvalho

**Affiliations:** 1 Faculty of Sport Sciences and Physical Education, University of Coimbra, CIDAF-Research Center for Sport and Physical Activity, Coimbra, Portugal; 2 Center for Neuroscience and Cell Biology, Center for Innovative Biomedicine and Biotechnology, University of Coimbra, Coimbra, Portugal,; 3 National Institute of Health, National Public Health Laboratory, Bissau, Guinea-Bissau,; 4 Bandim Health Project, Bissau, Guinea-Bissau,; 5 Steno Diabetes Center Copenhagen (SDCC), Herlev, Denmark,; 6 *Faculdade de Motricidade Humana (UTL-FMH)*, Exercise and Health by *Universidade Técnica de Lisboa*, Bissau, Guinea-Bissau,; 7 Institute of Hygiene and Tropical Medicine, New University of Lisbon, Lisbon, Portugal,; 8 National Diabetes Association (ANDD) and National Diabetes Clinic (CND), Bissau, Guinea-Bissau,; 9 Department of Endocrinology and Nephrology, University Hospital of Southwest Denmark, Finsensgade, Denmark,; 10 Institute of Interdisciplinary Research, University of Coimbra, Coimbra, Portugal

**Keywords:** Bioelectrical impedance, Omron, Tanita, body composition, Guinea-Bissau

## Abstract

**Introduction:**

the need to correctly measure and follow body composition as a simple disease prevention metric is important, especially where the healthcare infrastructures are poor. The variety of inexpensive devices available for this purpose is large. However, it is imperative to validate them in relation to the gold standard method, dual-energy absorptiometry X-ray (DEXA). In low-income countries, DEXA measurements aren't available. Thus, easy-to-use, and accurate devices are indispensable. In Guinea-Bissau, two relatively inexpensive, bioelectrical impedance scales, simple to use, are available. However, their accuracy has not been assessed in this setting. The study compares the level of agreement in measurements between, the Tanita® BC-545 and the Omron Karada Scan BF511, in adult volunteers.

**Methods:**

volunteers grouped for athletic and sports modalities at stadiums and sports facilities in Bissau were included. All anthropometric measurements were done in both devices. For statistical analysis, we created Bland-Altman plots to assess their level of agreement

**Results:**

the study included 274 participants, mean age 27.4 years, 214 (78%) males. For body fat, the median between the Omron and Tanita measures was 2.6 and the interquartile was 5.2. The Omron measured median body mass index, -0.3 kg/m^2^ and 0.8 kg/m^2^ of interquartile below that of the Tanita. For visceral fat, the Omron measured 1% of median and an interquartile of 2% above that of the Tanita. For skeletal muscle, the Omron median measured 11.3% and 11.4 % of interquartile below that of the Tanita. The intra-class correlation coefficient (ICC) for body fat (BF), body mass index (BMI) and skeletal muscle (SM) was 0.99 and for VF it was 1.00 on both devices.

**Conclusion:**

the results indicate a good level of agreement between the two devices. In resource-limited settings, the Omron is likely a reasonable substitute for more expensive body composition devices.

## Introduction

Body composition is an essential and vital element in monitoring of human health. It is an assessment tool used as a predictive and follow-up indicator for health conditions including, obesity, cardiovascular disease, type 2 diabetes and hypertension [1,2]. Anthropometric values, such as body mass index (BMI) and weight, are important factors to monitor over time in specific populations. However, these are not enough as they do not reflect the overall body fat mass or lean mass, for instance. The use of different devices to assess body composition such as, dual-energy X-ray absorptiometry (DEXA), magnetic resonance imaging (MRI), air-displacement plethysmography and bioelectrical impedance analysis (BIA) is unavoidable [3]. Dual-energy X-ray absorptiometry has been considered the gold standard for body composition assessment. It uses low dose X-rays for whole-body measurements of adipose tissue, fat % or lean mass. However, DEXA instruments are very expensive, and they are not portable, therefore, not suitable for field studies [4,5]. Simple, accurate, and non-invasive methods to assess body composition are needed in clinical communities, and research settings. The foot-to-foot bioelectrical impedance analysis (BIA) system may be a solution for these needs [1,4,6]. They are commonly used to estimate fat mass and fat-free mass [7]. These techniques, despite their low accuracy compared to the gold standard method, hold an advantage in field studies. They are less expensive, easy to carry around and noninvasive, but factors such as sex, age, ethnicity and stage of development (puberty) are some of the conditions that may affect BIA measurements. The Tanita BIA system has been commonly used and is a well-established system, convenient and practical for assessment in public health facilities [8]. Furthermore, the Omron Karada BIA system is a tetrapolar body composition monitor that incorporates both hand-to-hand and foot-to-foot electrical impedance technology, compared to some in the Tanita system, validated clinically and classified as a medical device by the Obesity Facts research group [9]. Guinea-Bissau, in West Africa, is a low-income country with very limited health and medical resources and technologies. The most accurate and reliable body composition analyzer devices in the country are the Tanita® BC-545 and the Omron Karada Scan BF511. These are available in the capital Bissau and without validation or conditions to do so against the gold standard DEXA. This study aimed to compare the levels of agreement between two BIA, the Tanita® BC-545 and the Omron Karada Scan BF511, in Guinean adults.

## Methods

**Study design and setting:** this small study is part of an ongoing large national diabetes screening study, to be reported elsewhere. Using a segmental body composition scale (Omron Karada Scan BF511), the distribution of body fat % and muscle mass could be determined. A variety of accepted methods are available for estimating body composition using BIA [7]. Bioelectrical impedance analysis has been shown reliable, portable, relatively inexpensive, convenient, and it is a non-invasive device to estimate body composition [10]. Bioelectrical impedance analysis (BIA) devices to assess body composition have been routinely used in health and fitness facilities, as well as in occupational health and research settings [3,11-13]. These devices measure the impedance to the flow of electrical current to estimate body composition, where greater electrical impedance is correlated with higher fat mass [11,14]. The method is based on the capacity of the hydrated tissues in the body to conduct electric current [15,16]. Bioelectrical impedance analysis devices include predictive equations of body fat developed in specific populations [17]. With multiple options for the assessment of body composition, it is important to provide evidence for the optimal use of these inexpensive tools. Furthermore, it is also important to define their accuracy and inter-changeability in terms of body sizes, and different populations across the world, including in sub-Saharan Africa [2]. Voluntary participants were recruited according to the inclusion criteria (below) and the measurements were performed first with Omron Karada Scan BF511 equipment and repeated with Tanita® BC-545 equipment within a few minutes. The comparison was made for agreement level assessment using the Bland-Altman method. Anthropometric measurements that included body weight, waist and hip circumference, height and body fat percentage were also conducted. The recruitment for this study was conducted at the Lino Correia stadium and at the 24 de Setembro stadium, as well as at the National School of Physical Education and Sport (ENEFD), in the capital of Bissau, Guinea-Bissau, where people were grouped for athletic, physical education classes, sports training and participating in sport modalities like basketball, handball and volleyball.

**Study population:** for this study we targeted students from ENEFD and sports facilities. The inclusion criteria were students aged 18 years old present at the Lino Correia and the 24 de Setembro stadiums and the ENEFD facilities that signed a consent form to participate, were included. The exclusion criteria any person that did not sign the consent to participate or had any disability that precluded them from joining the study, including participants under the age of 18, or those who were only present for a recreational activity but no link to the sports institutions, were excluded, as well as those who were not fasting on recruitment days, as this was part of a larger diabetes prevalence study. A review of the literature allowed us to identify previous studies that addressed similar comparisons between bioelectrical impedance scales, however, with smaller sample sizes. A study with a larger sample size was important for adequate statistical power and a high probability of detecting a true difference between the scales, if present. In addition, practical considerations such as the availability of resources and the time required for recruiting and measuring participants were considered. This study size was considered feasible within the resources available for the study.

**Data collection:** stature was measured by using a self-retracting ting measuring tape, steel, 3mtr/10ft, push-down blade lock for accurate measurements. Waist and hip were recorded using a flexible tape measure of 150 cm PRIMA, Mètre ruban 150cm PRIMA Meetlint. Blood pressure was measured using a Wellion WAVE professional Blood Pressure Monitor using the oscillometric method. Body composition was measured first with the Omron Karada Scan BF511 equipment, and then the Tanita® BC-545 equipment. Both measure the impedance to the flow of electrical current to estimate body composition, with greater electrical impedance correlating with higher fat mass. The method is based on the capacity of hydrated tissues to conduct electric current. The measurement was performed as per manufacturer´s guidelines. Briefly, information such as, age, gender and height taken by the ting measure tape were entered into the equipment and then the subject was allowed to stand on the metal footpads barefoot, grasping a pair of electrodes that were fixed on a handle with their arms extending in front of their chest on the scale after its calibration. The report provided was weight (kg), body fat (%, BF), visceral fat (%, VF), body mass index (kg/m^2^) and skeletal muscle (%, SM). Immediately after, the measurement with the Tanita® BC-545 equipment was performed, by entering date of birth, gender, height and the state of physical activity, in which one could choose whether the participant was an athlete, or physically active or sedentary.

Likewise, the participants were asked to stand on the metal footpads, barefoot, grasping a pair of electrodes fixed on a handle with their arms extended in front of their chest on the scale. The Tanita® equipment reported similar measures as the Omron. A clinical questionnaire was also applied to record information on alcohol and tobacco consumption, as well as systolic (sys_bp) and diastolic (dia_bp) blood pressure. Four repeated measures were performed in ten volunteers for ICC (Agreement Index or Intra-class Correlation Coefficient) evaluation, assessing BF, VF, BMI and SM in Tanita® BC-545. The same procedure was performed on the Omron Karada Scan BF511, to determine how consistent the measurements were when repeated (intra-class reliability). Standardized measurement procedures were controlled and their consistencies were ensured for both scales, including clear instructions for participants on how to use the scales correctly and adequate training for technicians who include and handle the two scales. Conditions that could influence the measurements were also identified and controlled, such as body hydration, level of physical activity, recent intake of food or liquids, or physical activity, before the measurement.

**Questionnaires:** clinical questionnaires were applied during the selection of the participants that included data on, anthropometry (variables such as height, weight and waist/hips ratio), socio-demographics (variables such as sex and age), bio impedance assessments (variables: BF, BMI, VF, SM), clinical (variables: systolic and diastolic blood pressure) and behavioral data (variables: alcohol and tobacco consumption).

**Statistical analysis:** analyses were performed using the STATA statistical software version 12.0 (Stata Corp, College Station, Texas). The assessment of whether the data were normally distributed was performed using the Shapiro-Wilk test, and reported as the median (interquartile range) for non-normally distributed variables and used the Mann-Whitney U test to compare variables between sexes. To assess levels of agreement between both measurement methods Bland-Altman plots were created, that calculated the mean differences and limits of agreement for BMI, body fat, skeletal muscle, and visceral fat, between the two devices [18]. Altman and Bland [18] suggested that in the absence of a gold standard method, it would be important to consider evaluating only the agreement of two measurements. Bland-Altman plot shows the differences between the two measurements against the mean of the two measurements, and hence, can provide a graphical overview of any systematic tendencies over the range of measurement. To calculate the ICC the ANOVA test was performed.

**Ethical considerations:** the informed written consent was obtained from all participant volunteers prior to participating in the two body composition assessments, using the Tanita® BC-545 and the Omron Karada Scan BF511. The study protocol was approved (Reference 015/CNES/INASA/2022) by the National Ethics Committee in health studies (CNES), National Institute of Health (INASA), Guinea-Bissau.

## Results

**Participants:** from January 30^th^ to February 16^th^ 2023, 274 participants were included. The time taken to make all the measurements on the two scales and the required fasting conditions for at least 8 hours prior to enrollment and the exclusion criteria, such as age, were limiting factors in the inclusion of more participants. Participants had a mean age of 27.4 (SD 7.2), 214 (78%) were male. The characteristics of the participants by gender are summarized in Table 1.

**Table 1:** demographic characteristics of participants by gender

Demographic characteristics	Male: median [inter-quartile range]	Female median [inter-quartile range]	P-value
	**N=214**	**N=60**	<0.0001
Age (years)	27 [24 - 31]	24 [20- 27]	<0.0001
Stature (m)	1.73 [1.68 -1.78]	1.62 [1.58- 1.68]	<0.0001
Weight (kg	63.5 [58.2- 69.4]	57.1 [50.3- 67.8]	<0.0001
Waist/hip ratio (m)	0.80 [0.78- 0.83]	0.75 [0.71- 0.79]	<0.0001
Blood pressure (sys_bp) (mmHg	132 [122- 143]	124.5 [133 – 134.5]	<0.0001
Blood pressure (dia_bp) (mmHg)	81 [75- 90]	80 [74.5- 85]	0.07
Smoking status	4.6% (10/214)	1.7% (1/60)	<0.0001
Smoking status	27.1% (58/214)	15% (9/60)	<0.0001

Sys_bp: systolic blood pressure; Dia_bp: diastolic blood pressure

**Figure 1:**
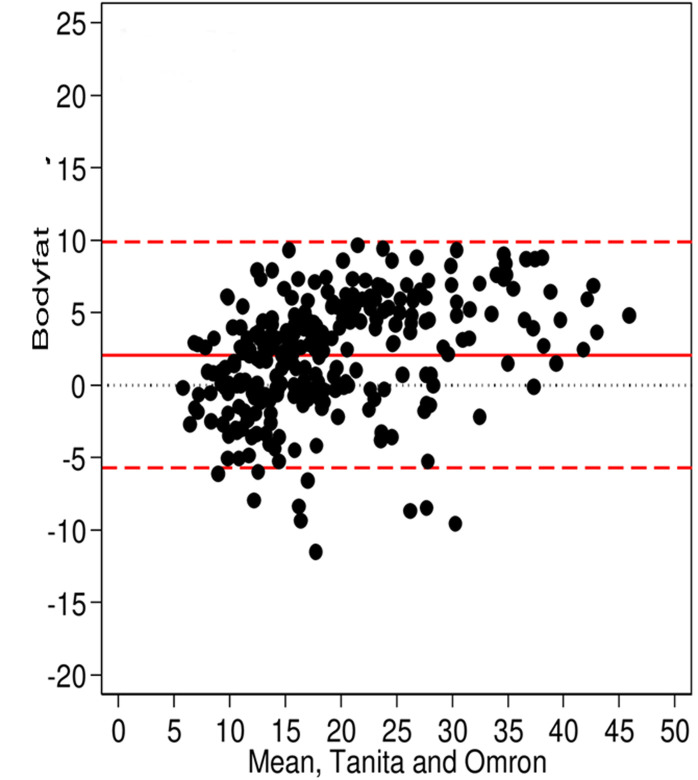
a Bland-Altman plot of body fat % measured with the Tanita® BC-545 and the Omron Karada Scan BF511

**Figure 2:**
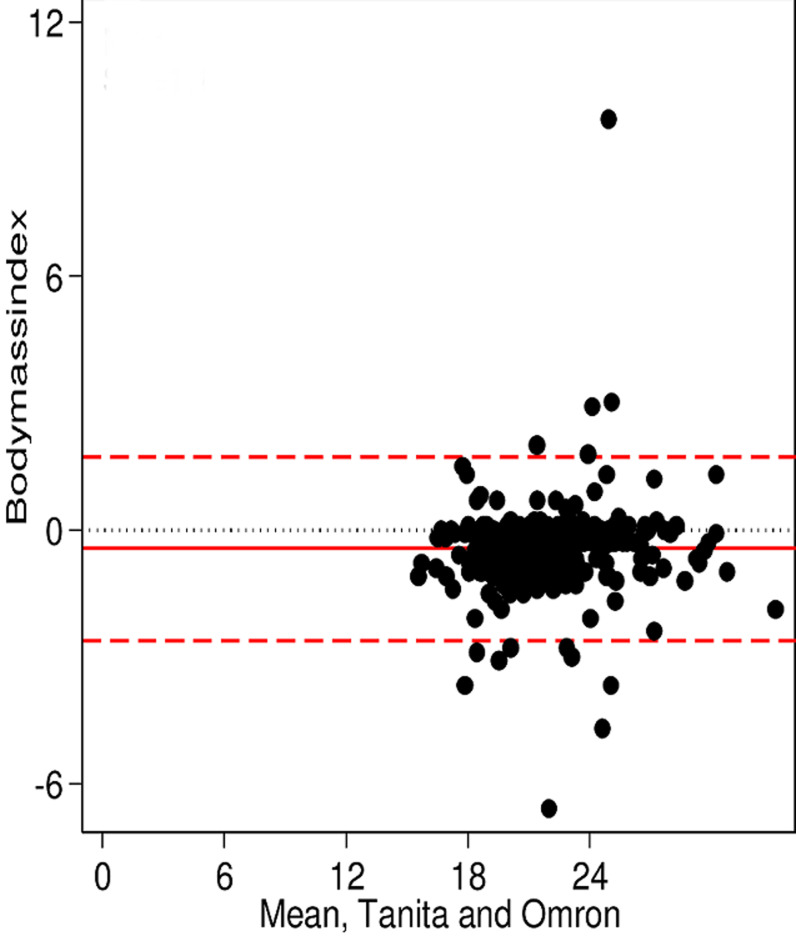
a Bland-Altman plot showing body mass index measured using the Tanita® BC-545 and the Omron Karada Scan BF511

**Figure 3:**
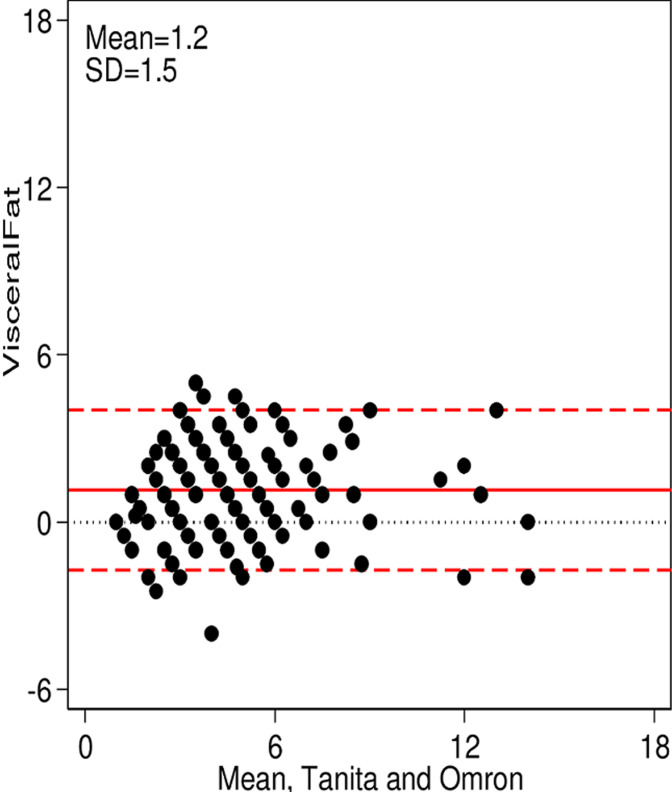
a Bland-Altman plot of visceral fat mass measured using the Tanita® BC-545 and the Omron Karada Scan BF511

**Figure 4:**
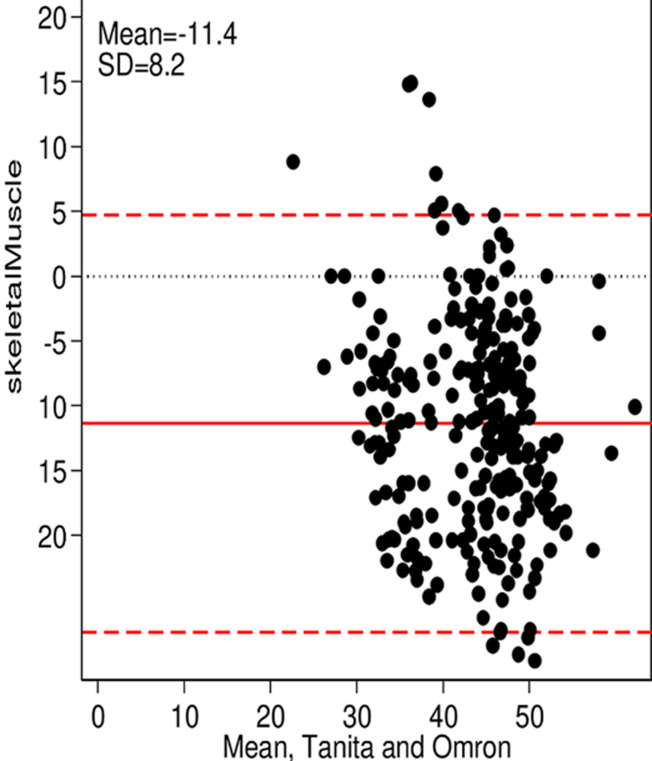
a Bland-Altman plot of skeletal muscle mass measured using the Tanita® BC-545 and the Omron Karada Scan BF511

**Main results:** the Bland-Altman plots showed an overall good agreement between the measurements from the two BIA scales and there were also no obvious systematic trends. The results presented in Figure 1 evaluated and compared body fat (BF %) measurements, between the Omron Karada Scan BF511 and the Tanita ® BC-545. The median (50° percentile) of the difference between the two measurements was 2.6%, with interquartile of 5.2%. For body mass index (BMI) measurement, the agreement is very good comparing both scales. The Omron Karada Scan BF511 measures a median of -0.3 Kg/m^2^ below those of the Tanita® BC-545 with an interquartile of 0.8, the mean difference was shown in Figure 2. Furthermore, Figure 3 shows the agreement in the measurements verified for visceral fat (VF %) mass. The Omron Karada Scan BF511 measures a median of 1% above those of the Tanita® BC-545, with an interquartile of 2%. While Figure 4 represents the measurements for skeletal muscle (SM %). The Omron Karada Scan BF511 values for skeletal muscle mass are a median of -11.3% below those observed by the Tanita® BC-545, with an inter-quartile 11.4%. Moreover, the ICC measurement was 0.99 for BF, BMI and SM and 1.00 for VF, when comparing 4 repeated measures, in 10 different participants with the Tanita® BC-545 and the Omron Karada Scan BF511, showing strong consistency of repeated measures.

## Discussion

The aim of this study was to compare the level of agreement between the Omron Karada Scan BF511 and the Tanita® BC-545 BIA equipment in a small sample of young students from the capital Bissau, in Guinea-Bissau. Our results overall demonstrated good levels of agreement between the Tanita® BC-545 and the Omron Karada Scan BF511 in the measurements of BF (%), BMI (kg), VF (%) and SM (%). Furthermore, the ICC revealed strong consistency of the measured values. Many studies have compared and evaluated the agreement of measurements between Tanita equipment such as the Tanita® BC-532 [4], the Tanita TBF-401 [10], the Tanita® BC-532 [11], the Tanita BF-625 [19] and the DEXA, considered the gold standard. Some report significant differences in the measurement of body fat, as is the case of Lazzer *et al*. [19], LaForgia *et al*. [11] and Samouda *et al*. [4], who advise the calibration of the equipment before using it in a population in which it has not been previously validated Sung *et al*. [10] confirmed an agreement between the Tanita TBF-401 and the DEXA in validated measurements in a Chinese population, despite an underestimation of the body composition values. Jensky-Squires *et al*. [16] advanced with a comparison of % BF between the Omron HBF-360 and the Bod-eComm scales against the DEXA and the differences found were not significant. Still, other studies conducted by Xu *et al*. [20], comparing the Tanita® BC-532, the Omron HBF-359 and the DEXA, in a Chinese adult population in measurements of BF, VF, SM, demonstrated a high correlation of the two scales with the DEXA gold standard.

The Omron BF511 scale has been compared with other methods, such as the skinfold thickness measurement using the caliper Best II. K501, bioimpedance, using devices Bodystat 1500 and Omron BF300 by Brtková *et al*. [6], in the evaluation of body fat. After constructing Bland-Plots, the measurements of the Omron BF511 were comparable and in agreement with the other methods, such as Bodystat 1500, Omron BF300 and skinfolds. Randhawa *et al*. [14] compared measurements between the Tanita® BC-418, Tanita® TBF-314 and the Omron HBF-306. They did not find significant differences in the measurements with the three scales [14]. These results were consistent with those of Vasold *et al*. [21] that in the measurement of free fat mass, using the Tanita® BC-534 and the Omron HBF-510 resulted in high reliability and validity. These results agree and confirm the results obtained in this study. However, Brtková *et al*. [6], with 52 participants included, concluded that the Omron BF511 scale did not provide results that could be considered sufficiently accurate for the purposes of research and could not be used for research and proposes, suggesting a larger study with more power (n100 - 300) would be needed to confirm the conclusion. Thus, the present study, comparing the Omron BF511 with the Tanita® BC-545, a good agreement was observed, and the differences were small, using a sample of 274 adult participants, well above the number of participants recommended by Brtková *et al*. [6]. Moreover, when the ICC was calculated the results were almost perfect (0.99 for BF, BMI, SM and 1.0 for VF), translating high reliability of measures when comparing the Tanita® BC-545 to the Omron Karada Scan BF511.

Assessment of body composition provides an important set of measurements that can be used in the management of human health in general and especially in people at risk of obesity, and chronic diseases, as well as in the field of weight management, fitness and athletics. But for a correct measure and a reliable equipment, it is necessary to have proper guidelines. This study aimed to compare the values and their agreement between the two BIA scales available in Bissau, Guinea-Bissau in adult participants who attended physical activity and athletics institutions. Young Guineans aged 18 or older of both sexes were included. All participants were physically active (they routinely practice sports) and all appeared to be healthy. The measurements were made in a research environment, and the methodologies used during these measurements meet the criteria of those used in similar studies. The observed results revealed good agreement between the two BIA scales, in the Guinean-Bissau population. Due to the low resource nature of many countries on the African continent, these two scales may be a good choice, and it´s use easily implementable. However, it is important to note that a calibration may be required for the use of the same scales in other populations. One of the limitations of this study is the unavailability of the gold standard method, the DEXA in Guinea-Bissau, to assess and compare the accuracy of the Omron Karada Scan BF511. It should also be noted that we made our comparisons in a selected group of young and physically active individuals. Generalization to the overall population, including obese individuals, should therefore be done with some caution.

## Conclusion

Our results showed that the measurement of body composition, using two different and relatively inexpensive BIA scales, the Tanita® BC-545 and the Omron Karada Scan BF511, available in Guinea-Bissau, a low-income country, were in overall good agreement when measuring BF, VF, SM and BMI. These results are important for future field studies in low- or middle-income countries, where this type of equipment is often lacking, due to cost or the need for equipment validation.

### What is known about this topic

- *High-accuracy BIA devices for clinical, fitness and investigative purposes to determine body composition have been used in many studies outside Africa;*
- *Several BIA devices have been validated against the gold standard, DEXA, in determining body composition, even in research settings;*
- *Some studies concluded that BIA does not provide results sufficiently accurate for research purposes, thus confirmation of accuracy in larger studies is needed*.

### What this study adds

- *This study compares two BIA scales in a low-income country setting, the Omron Karada Scan BF511 and the BIA Tanita® BC-545, results demonstrated good agreement;*
- *Body composition was assessed for the first time in Guinea-Bissau, in a clinical research setting and the results present confidence and accuracy;*
- *In a sample of 274 young participants, the Omron Karada Scan BF511 provided perfect agreement level, translating high reliability in the results compared with the Tanita® BC-545*.
